# Dietary inorganic nitrate: From villain to hero in metabolic disease?

**DOI:** 10.1002/mnfr.201500153

**Published:** 2015-08-26

**Authors:** Ben McNally, Julian L. Griffin, Lee D. Roberts

**Affiliations:** ^1^ Elsie Widdowson LaboratoryMedical Research Council – Human Nutrition ResearchCambridgeUK; ^2^ Department of Biochemistry and Cambridge Systems Biology CentreUniversity of CambridgeCambridgeUK

**Keywords:** Adipose Tissue, Diabetes, Dietary inorganic nitrate, Metabolism, Metabolic disease, Nitric oxide, Obesity

## Abstract

Historically, inorganic nitrate was believed to be an inert by‐product of nitric oxide (NO) metabolism that was readily excreted by the body. Studies utilising doses of nitrate far in excess of dietary and physiological sources reported potentially toxic and carcinogenic effects of the anion. However, nitrate is a significant component of our diets, with the majority of the anion coming from green leafy vegetables, which have been consistently shown to offer protection against obesity, type 2 diabetes and metabolic diseases. The discovery of a metabolic pathway in mammals, in which nitrate is reduced to NO, via nitrite, has warranted a re‐examination of the physiological role of this small molecule. Obesity, type 2 diabetes and the metabolic syndrome are associated with a decrease in NO bioavailability. Recent research suggests that the nitrate‐nitrite‐NO pathway may be harnessed as a therapeutic to supplement circulating NO concentrations, with both anti‐obesity and anti‐diabetic effects, as well as improving vascular function. In this review, we examine the key studies that have led to the re‐evaluation of the physiological function of inorganic nitrate, from toxic and carcinogenic metabolite, to a potentially important and beneficial agent in the treatment of metabolic disease.

**Abbreviations**
BAT: brown adipose tissue
cGMP: cyclic guanosine mononucleotide phosphate
CVD: cardiovascular disease
eNOS: endothelial nitric oxide synthase
iNOS: inducible nitric oxide synthase
NADPH: nicotinamide adenosine dinucleotide phosphate
NO: nitric oxide
NOS: nitric oxide synthase
T2DM: type 2 diabetes mellitus
UCP‐1: uncoupling protein 1
WAT: white adipose tissue
XOR: xanthine oxidoreductase

## 1 Introduction

Until recently dietary inorganic nitrate was thought to be hazardous to human health. In 2006, the International Agency for Research on Cancer (IARC) concluded that it was probable ingested nitrate was carcinogenic in conditions that promoted endogenous nitrosation 1. The final report, not published until 2010 2, highlighted how ingested nitrate and nitrite could trigger carcinogenesis through the formation of deleterious species such as *N*‐nitroso compounds in the presence of nitrosatable compounds. The conclusions of these reports were based on epidemiological studies 3, 4, 5, 6, 7, and biochemical theory building on the initial finding that secondary nitrosamines are highly carcinogenic 8. However, a recent review has been highly critical of these studies 9, highlighting numerous investigations which do not find an association between nitrate intake and gastric cancers 10, 11, 12, as well as confounding issues in the methodology of previous studies which utilised doses of nitrate far in excess of physiological sources.

Nitrate is an important constituent of our diet. Sources of dietary nitrate intake include processed meats, fruits and vegetables. In addition, nitrates and nitrites are often added as a preservative to meats, including sausages, hot dogs and bacon, and were initially suggested to be contributing to the risk to human health posed by preserved and processed meats 13, 14 leading to tight controls on their use. However, of all dietary sources of nitrate it is vegetables that contain the highest concentration, a food group well documented to reduce the risk of obesity and cardiometabolic disease 15, 16, 17, 18. The concentration of nitrate in numerous fruits and vegetables has been well characterised by Hord et al. (Table 1) 19. Green leafy vegetables, such as celery, spinach, lettuce and rocket, contain the highest nitrate content.

**Table 1:** The nitrate content of food types, showing the disparity between vegetables, fruits and cured meats, as well as the intra‐variation within vegetables

Amount of nitrate (mg/100 g fresh weight)	Food type
Less than 20	Fruits including banana and orange
	Cured meats including bacon, ham and hot dogs
	Vegetables including onion, pepper, pea, asparagus, mushroom
20–50	Vegetables including broccoli, carrot, cauliflower and cucumber.
50–100	Vegetables including turnip and cabbage
100–250	Vegetables including leek and fennel
More than 250	Vegetables including spinach, lettuce, celery and rocket

Table adapted from Hord et al. 19.

Since the concentration of nitrate and nitrite in vegetables is so much higher than processed meats, it appears unlikely that nitrates and nitrites are the active agents in the risk to health associated with processed meats. In fact, the World Health Organisation acceptable daily intake for nitrate is 0–3.7 mg/kg. This figure is vastly exceeded (approximately 550%) by a diet relatively rich in fruit and vegetables high in nitrate 19. Even one portion of spinach can exceed the acceptable daily intake for nitrate. Considering that green leafy vegetables have been consistently shown to offer protection against obesity and cardiometabolic disease in numerous epidemiological studies, the view of nitrate and nitrite must be reassessed. With the identification of an endogenous metabolic pathway catalysing the reduction of nitrate via nitrite to nitric oxide (NO), the nitrate‐nitrite‐NO pathway 20, 21, 22, we have seen a paradigm shift in our understanding of the physiological function of nitrate, in particular with regards to ischaemia‐reperfusion injury, stroke, gastric ulceration and, most recently, obesity and aspects of the metabolic syndrome 23, 24.

Here, we discuss the therapeutic potential of inorganic nitrate, as a means of providing bioavailable NO, to treat obesity, diabetes and the metabolic syndrome.

## 2 Nitrate‐nitrite‐NO pathway

**Figure 1:**
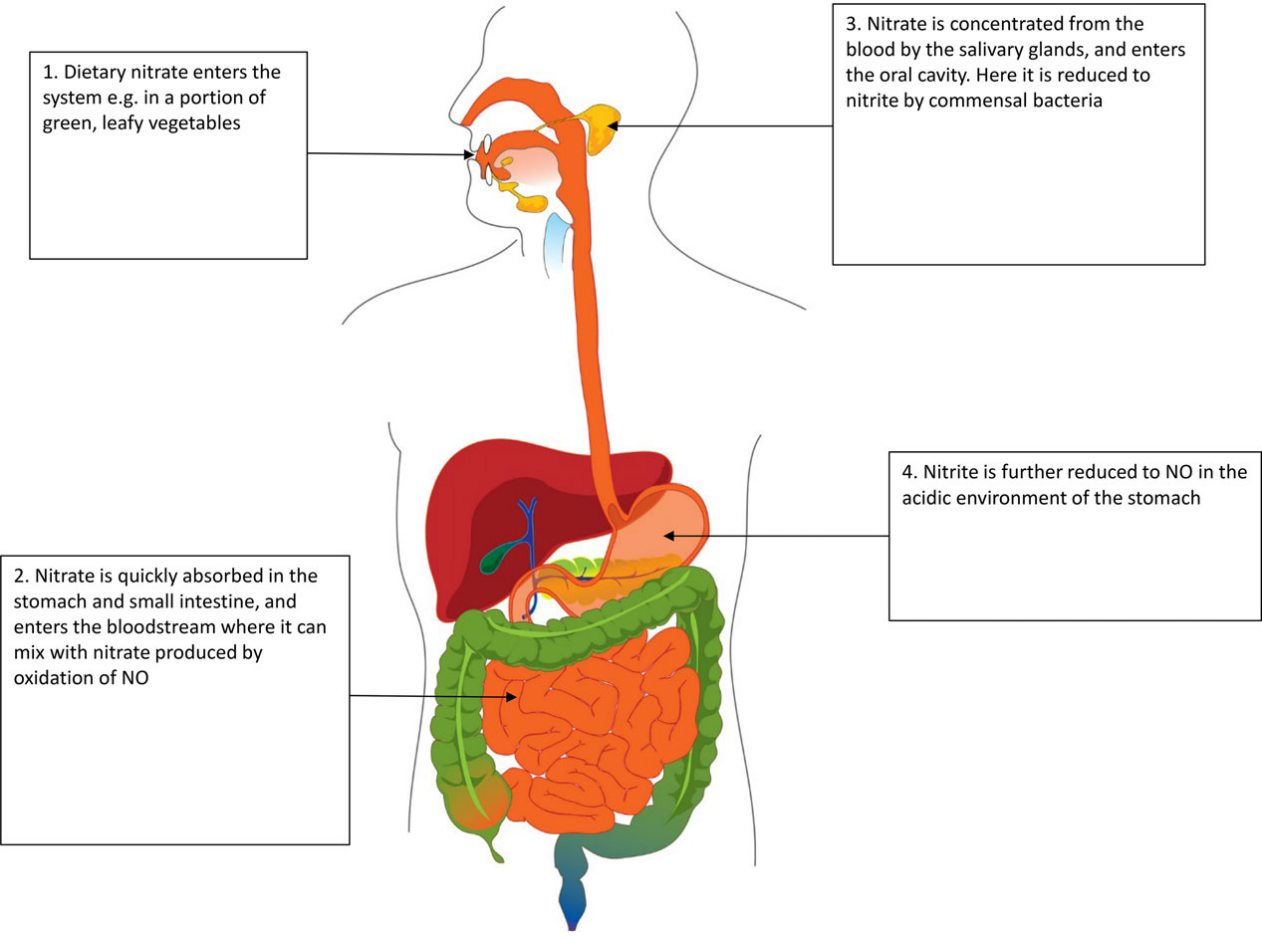
Dietary nitrate is sequentially reduced to nitrite and then NO via the enterosalivary pathway. Once nitrate is ingested, it is absorbed into the bloodstream in the upper gastrointestinal tract. Here it can mix with nitrate produced by the oxidation of NO and nitrite. This nitrate can then continue along the enterosalivary pathway to be reduced to NO by commensal bacteria in the oral cavity. Further, nitrate could enter cells such as adipocytes (where it may be reduced to NO to bring about systemic changes), or it may be excreted by the kidneys.

NO is a highly bio‐active molecule, signalling via soluble guanylyl cyclase and cyclic guanosine mononucleotide phosphate (cGMP) to bring about a host of physiological functions 25. Initially, NO was identified as endothelial‐derived growth factor, highlighting its role as a potent vasodilator 26, 27. Further study confirmed fundamental roles for NO/cGMP signalling in the nervous system and the immune system 28, 29, 30. Canonically, NO is synthesised by a family of enzymes termed nitric oxide synthases (NOSs), which consist of three isoforms: neuronal nitric oxide synthase (NOS1), inducible nitric oxide synthase (iNOS, NOS2) and endothelial nitric oxide synthase (eNOS, NOS3) 31. These enzymes produce NO through the metabolism of l‐arginine to l‐citrulline 32. However, a second pathway was subsequently described in vivo identifying the serial reduction of nitrate to nitrite and then to NO 20, 21, 22. This mechanism has been termed the nitrate‐nitrite‐NO pathway and was initially thought to be purely non‐enzymatic. Nitrate from the diet enters the enterosalivary pathway 24 and is then absorbed in the upper gastrointestinal tract entering the blood and raising plasma nitrate levels. A large proportion (approximately 65%) of this nitrate is excreted in the urine 33, but approximately 25% is transported into the salivary glands and concentrated into the saliva (Fig. 1) 22, 34. Commensal bacteria present in the mouth then use the nitrate as an alternative electron acceptor during respiration, reducing the anion to nitrite 35. The nitrite in the saliva then enters the acidic environment of the stomach, where it is reduced to NO via a protonated intermediate, nitrous acid 20, 36. The pathway is enhanced by reducing agents found in the diet, such as vitamin C and polyphenols 37, 38, highlighting the complex interactions between dietary nitrate and additional compounds found as dietary constituents.

Following the description of the non‐enzymatic physiological reduction of nitrate through nitrite to NO, work conducted by Jansson et al. identified an enzymatic process functioning to reduce nitrate to NO in mammalian tissue, with xanthine oxidoreductase (XOR) postulated as the enzyme responsible 39. Previous studies had suggested that mammalian cells and tissues were incapable of reducing nitrate 40, but studies, inspired by the earlier identification of nitrate reductase activity of milk XOR 41, led to the in vitro determination of nitrate reduction by mammalian XOR 42, 43. Jansson et al. built on this work, demonstrating nitrate reductase activity in a range of rodent and human tissues, which can be inhibited by allopurinol, an inhibitor of XOR 39. This enzymatic pathway provides an alternative means for the generation of nitrite and NO from dietary nitrate, distinct from the enterosalivary pathway. Jansson et al. also suggested that the enzymatic reduction of nitrate may be as important as the enterosalivary pathway for the production of NO from nitrate, in rodents at least. Plasma nitrite was found to increase in both germ‐free and wild‐type mice to a similar extent, implying a non‐bacterial mechanism of nitrate reduction capable of accounting for the loss of nitrite from enterosalivary pathways. In contrast, however, Govoni et al. demonstrated that the use of an antibacterial mouth wash in humans reduced the concentrations of plasma nitrite, and may reinforce the importance of the enterosalivary pathway. This discrepancy may be due to a lower concentration of nitrate in the saliva of rodents [44]. Currently, the exact contribution of the enterosalivary and enzymatic pathways to the reduction of nitrate to NO in vivo remains undefined.

A number of additional physiological sources of nitrate reduction have been identified. Cosby et al. demonstrated that infusions of nitrite into the forearm brachial artery resulted in rapid formation of erythrocyte iron‐nitrosylated haemoglobin 45, the NO bound form of haemoglobin. This reaction was inversely proportional to oxyhaemoglobin saturation, highlighting deoxyhaemoglobin as the driver of nitrite reduction to NO. Deoxymyoglobin can also reduce nitrite to NO, at a rate approximately 36 times faster than deoxyhaemoglobin 46. The deoxymyoglobin‐mediated reduction of nitrite to NO has been shown to be important in mitochondrial respiration, cardiac energetics and hypoxic vasodilation 46, 47, 48. Aspects of the mitochondrial respiratory chain have also been implicated in nitrite reduction 49, 50, 51. Nohl et al. used the nitrosylation of deoxyhaemoglobin to follow nitrite reduction to NO by respiring mitochondria. Treatment with mitochondrial complex inhibitors identified ubisemiquinone, as part of the ubiquinone/bc_1_ couple (complex III), as essential in the reduction of nitrite to NO by mitochondria 49. Castello et al. also demonstrated that cytochrome c oxidase (complex IV) could also reduce nitrite to NO in hypoxic conditions 50.

NO has a short half‐life (0.05–1.18 ms in human blood), meaning that it can only act locally in an autocrine and paracrine manner to bring about physiological changes 47, 52. However, the half‐lives of both nitrate and nitrite are relatively long in comparison (110 s and 8 h, respectively, in human blood) 52. Therefore, nitrate and nitrite are more stable and can circulate in the blood, where they may be reduced via the nitrate‐nitrite‐NO pathway back to NO, and therefore function in an endocrine manner.

While on the surface it appears that the nitrate‐nitrite‐NO pathway is a redundant mechanism for generating NO, the recycling of nitrate and nitrite in fact provides an important method of producing NO in conditions where NOS enzyme activity is compromised. The NOS enzymes require oxygen to produce NO, therefore NO production via the canonical l‐arginine‐NO pathway is impaired in hypoxic conditions 24. In contrast, the nitrate‐nitrite‐NO pathway is capable of reducing nitrate to NO in low oxygen concentrations, and may indeed be more efficient in hypoxia 48, 49, 50, 53, 54. In the case of adipose tissue, the expression of XOR is stimulated by low oxygen availability 55. This finding highlighted the possibilities of using nitrate and nitrite as therapeutics to treat disorders in which NO synthesis is compromised.

However, the enzymatic reduction of nitrate has been observed to occur in both hypoxic and normoxic conditions, implying a role beyond that of a compensatory mechanism for compromised eNOS activity 39. This was further supported by work from Huang et al., demonstrating the importance of XOR in normoxic reduction of nitrate in germ‐free mice 56. Studies conducted by Roberts et al. also demonstrated that nitrate treatment could in fact stimulate XOR expression in normoxic adipocytes, albeit to a lesser degree than in hypoxia, and highlighted that XOR‐mediated nitrate reduction had downstream functional effects in normoxic conditions 55.

## 3 Dietary nitrate and the metabolic syndrome

The metabolic syndrome is a cluster of risk factors for cardiovascular disease (CVD) and type 2 diabetes mellitus (T2DM). These factors include obesity, dyslipidaemia, hypertension and insulin resistance 57, 58. The prevalence of the metabolic syndrome is reaching epidemic proportions. The World Health Organisation estimates 347 million people worldwide suffer from diabetes mellitus 59. Of these cases, 90% are associated with T2DM, with obesity as the leading risk factor. However, the identification of the underlying molecular mechanisms that unite the aspects of the metabolic syndrome are challenging due to the diffuse and complex nature of the disease, which integrates peripheral insulin resistance, visceral obesity and CVD. For example, many mechanisms have been postulated as the root of insulin resistance, and it is likely that no one pathway is responsible for the dysfunction of insulin signalling. One of the most accepted causes is the inappropriate accumulation of lipids within peripheral tissues, known as lipid‐induced insulin resistance 60. This mechanism was initially suggested by Randle et al. in a study showing intracellular glucose‐6‐phosphate increases as a result of incubating rodent heart with fatty acids 61. They postulated that this was a result of decreased glycolysis. However, further studies have indicated that lipid‐induced insulin resistance may arise due to impaired insulin signalling and subsequent reduced glucose uptake 62, 63.

In recent years, the perturbation of NO synthesis and signalling has emerged as a potential modulator of both cardiovascular morbidity and metabolic dysfunction in both rodent models and humans 64, 65. In humans, variants in the eNOS gene are associated with aspects of the metabolic syndrome, giving genetic susceptibility to T2DM and insulin resistance 65. Furthermore, obese individuals were found to have a decreased capacity for NO production 66. Mice lacking the gene for eNOS develop a metabolic syndrome‐like phenotype, characterised by hypertension, glucose intolerance and insulin resistance 67, 68. Furthermore, eNOS‐null mice also display dyslipidaemia, with elevated circulating levels of cholesterol, triglycerides, free fatty acids and leptin accompanied by a 30–40% increase in visceral fat. The widespread effects of eNOS disruption highlight the role of NO as a possible unifying mechanism that underpins the metabolic syndrome.

Following the characterisation of eNOS‐null phenotypes, Carlstrom et al. sought to restore NO signalling in eNOS‐null mice via dietary nitrate supplementation and subsequent metabolism through the nitrate‐nitrite‐NO pathway 69. Seven weeks of dietary nitrate treatment decreased weight gain, visceral fat and plasma triglyceride concentrations, compared to untreated controls. Treatment with nitrate also improved glucose homeostasis, lowering fasting blood glucose concentrations and glycosylated haemoglobin, and improving glucose tolerance, as highlighted by glucose tolerance tests. Additionally nitrate administration reduced blood pressure independently of NOS. Interestingly, the effects of nitrate on the metabolic syndrome‐like phenotype of eNOS‐deficient mice were achieved with a concentration of nitrate corresponding to a daily dietary intake of 100–300g of a nitrate‐rich vegetable, such as spinach.

The work of Carlstrom et al. provided functional justification for the use of nitrate to treat impaired NO synthesis and highlighted the effects of dietary nitrate on, not only one, but multiple risk factors underlying the metabolic syndrome. However, while this study showed the potential of dietary nitrate as an anti‐obesity and anti‐diabetic agent on a phenotypic level, the mechanisms driving these effects were yet to be elucidated.

## 4 Nitrate and adipose tissue metabolism

Obesity is characterised by inappropriate accumulation of white adipose tissue (WAT) and an increase in adipocyte size. WAT stores energy as lipid which can then be released via lipolysis when required 70. White adipocytes have a unilocular lipid droplet that takes up the vast majority of the cell and few mitochondria reflecting its low demand for energy 71.

In contrast, brown adipose tissue (BAT) is more metabolically active and utilises lipids as an energy source in a process termed non‐shivering thermogenesis 72. Brown adipocytes are small multilocular cells, characterised by a large number of mitochondria and synthesis of uncoupling protein 1 (UCP‐1). In the mitochondria, UCP‐1 acts to uncouple electron transport from ATP production, leading to the generation of heat, by increasing proton leak across the mitochondrial membrane 73. Since thermogenesis is associated with an increase in lipolysis and fatty acid catabolism, via β‐oxidation, to supply fuel for the TCA cycle of the uncoupled mitochondria, it may have anti‐obesity properties, restoring the defective energy balance in obese individuals. The adipose‐specific transgenic expression of UCP‐1 prevents obesity in mice 74, whilst the genetic ablation of BAT in mice results in obesity 75. However, while thermogenesis appears to have therapeutic potential for the treatment of obesity, it is limited by the small amount of BAT present in adult humans, and is negatively correlated with age and adiposity 76.

Interestingly, a small number of cells within WAT were found to express UCP‐1 upon cold exposure 77. These cells were termed “beige” or “brite” cells and exhibit a gene expression profile distinct from either white or brown adipocytes 78. The identification of beige adipocytes within WAT depots has increased interest in the utilisation of thermogenesis and increased energy expenditure to treat obesity. Beige cells have a low basal expression of UCP‐1 but retain the capacity to greatly increase the expression of brown adipocyte‐specific genes leading to increased respiration, mitochondrial biogenesis and fatty acid β‐oxidation. The switch from a white adipocyte‐like to a brown adipocyte‐like phenotype is known as the browning response and has anti‐obesity and anti‐diabetic effects 74, 79

Numerous activators of the browning response have been identified, including cardiac natriuretic peptides, fibroblast growth factor 21, irisin and β‐aminoisobutyric acid 80, 81, 82, 83, 84. Recent studies have suggested that the anti‐obesity effects of inorganic nitrate may function, in part, by inducing the browning response 55.

Dietary nitrate is known to increase the circulating concentration of cGMP in humans 85, while a diet deficient in nitrate decreases the steady‐state concentration of cGMP in certain tissues 86. cGMP has a profound effect on the regulation of energy metabolism in adipose tissue by activating adipocyte differentiation and lipolysis 87, 88, 89. In 2013, Mitschke et al. identified cGMP as a small molecule capable of signalling via protein kinase G to stimulate the browning response in WAT 90. In response to these previous studies Roberts et al. hypothesised that nitrate may induce the browning of WAT 55.

**Figure 2:**
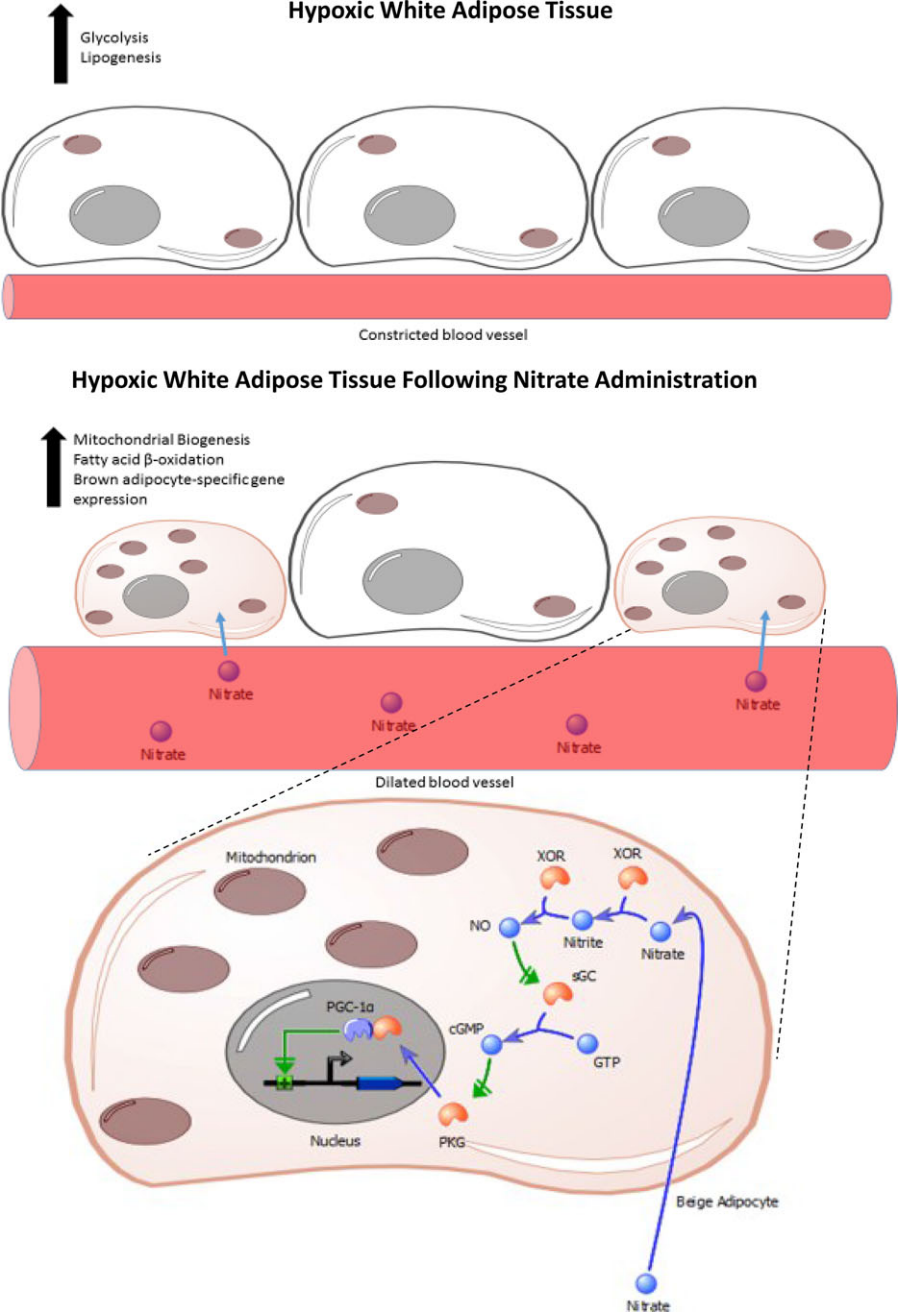
The effects of dietary inorganic nitrate on white adipose tissue. Dietary nitrate stimulates the browning response in hypoxic white adipose tissue. Nitrate is taken up from the blood by beige adipocytes where it triggers an increase in mitochondrial biogenesis, fatty acid β‐oxidation and brown adipocyte‐specific gene expression. The metabolic activity of the adipocytes is increased, raising the rate of thermogenesis. The mechanism by which nitrate activates browning relies on cGMP‐PKG signalling, followed by increased expression of the transcriptional activator PGC‐1α. PKG, protein kinase G.

Roberts et al. demonstrated that inorganic nitrate increased brown adipocyte‐specific gene expression, including UCP‐1, in WAT both in vitro and in vivo 55. The authors further characterised the brown adipocyte‐like phenotype induced by nitrate using respirometry and stable isotope flux labelling analysis to confirm an increase in oxygen consumption and flux through the fatty acid β‐oxidation pathway. Roberts et al. went on to elucidate the mechanisms underpinning the nitrate‐induced browning effect (Fig. 2). Both the NO scavenger 2‐phenyl‐4,4,5,5‐tetramethylimidazoline‐1‐oxyl 3‐oxide and siRNA knockdown of XOR inhibited the nitrate‐mediated induction of brown adipocyte‐specific gene expression in primary adipocytes. The immediate reduction product of nitrate, nitrite, was also identified as an activator of the browning response. Using additional pharmacological inhibitors nitrate was found to signal downstream of NO via a pathway involving cGMP and protein kinase G. These findings lead the authors to conclude that nitrate functioned through the nitrate‐nitrite‐NO pathway and subsequent cGMP signalling to activate the browning response in adipose tissue, underlining the importance of NO in mediating the browning effects of nitrate.

As previously mentioned, the nitrate‐nitrite‐NO pathway acts to complement the oxygen‐dependent NO synthases and utilises nitrate as a substrate in hypoxic conditions, thus providing NO in situations in which NO synthases are compromised 24. Roberts et al. showed that both primary adipocytes and WAT from rats exposed to low oxygen levels had increased levels of XOR expression, which was further increased by nitrate treatment. In keeping with these concepts, the authors found that nitrate‐induced browning was augmented by hypoxia (Fig. 2). This is particularly relevant given that the adipose tissue of obese mice and humans is characterised by hypoxia 91, 92, 93. The oxygen concentrations utilised in this study mimicked oxygen levels found in obesity 93, thus nitrate may be an effective means of inducing the browning response in adipose tissue to treat obesity, T2DM and the metabolic syndrome.

Carlstrom et al.’s work highlighted how dietary nitrate can bring about a reduction in body weight and dyslipidaemia, while increasing glucose tolerance, on a phenotypic level 69. The studies on the browning response show clearly how nitrate can readjust the energy imbalance in obesity, enhanced by the hypoxic conditions in the adipose of obese subjects. The catabolism of lipids through increased fatty acid beta‐oxidation provides a means to lower dyslipidaemia and alleviate aspects of lipid‐induced insulin resistance in type 2 diabetics. Importantly, dietary nitrate appears to target multiple aspects of the metabolic syndrome.

## 5 Nitrate and hypertension

Dietary nitrate has a profound effect on hypertension, a major risk factor for CVD and another strand of the metabolic syndrome 94. High blood pressure is defined as 140/90 mmHg 95. Uncontrolled hypertension can increase the risk for CVD by nearly three times 96, while controlling hypertension reduces the risk of CVD 94, 97, 98, 99. Furthermore, according to the Centers for Disease Control and Prevention, approximately 67% of diabetic patients also have hypertension 100.

Endothelial dysfunction is a significant determinant of hypertension 101. The loss of bioavailable NO has been implicated in endothelial dysfunction and the development of CVD 102, 103. Therefore, raising the levels of NO may have a beneficial impact on endothelial function and subsequent CVD. The potential for dietary nitrate to provide the much‐needed supply of NO in these pathological situations has been examined in a number of recent studies in humans 104. Ingestion of a single dose of beetroot juice, which contains a high concentration of nitrate, lowered both systolic and diastolic blood pressure 105, with the greatest effect after 3 h of nitrate consumption, concomitant with the highest concentrations of plasma nitrite and increased circulating cGMP concentrations. This lag may point towards nitrate as the causative agent and appears to resemble the time taken for nitrate to be reduced to nitrite, suggesting that the effects of dietary nitrate on blood pressure are mediated via the nitrate‐nitrite‐NO pathway. A study by Kenjale et al. further demonstrated that dietary nitrate reduces blood pressure in patients with peripheral artery disease 106, while nitrate has also been observed to lower blood pressure in hypertensive individuals 107. A recent study by Kapil et al. investigated the long‐term effects of daily doses of nitrate over a 4‐week period 108. Sustained dietary nitrate intake was found to reduce blood pressure, improve endothelial function and reduce arterial stiffness without any indication of tachyphylaxis over the 4‐week period. Together, these studies highlight the potential of dietary nitrate as a therapeutic for patients with hypertension.

**Figure 3:**
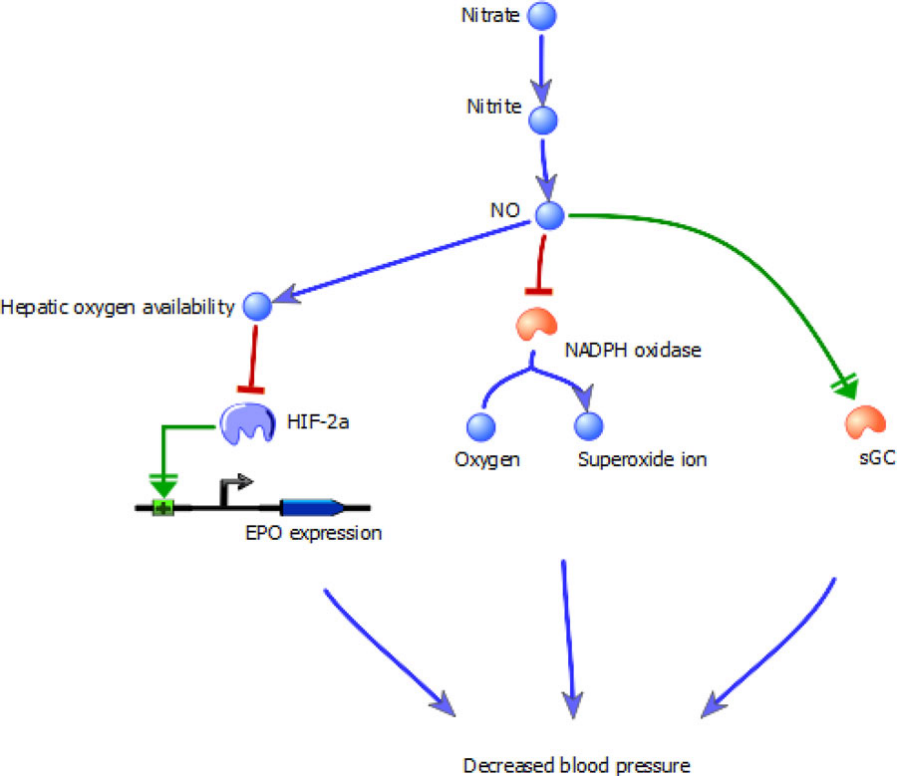
The three proposed mechanisms through which inorganic nitrate reduces blood pressure. Nitrate is converted to NO via the enterosalivary pathway or the XOR‐catalysed nitrate‐nitrite‐NO pathway. NO can then activate soluble guanylyl cyclase (sGC), reducing blood pressure in the canonical manner. Further, it can inhibit superoxide production by NADPH oxidase, a vasoconstriction pathway stimulated by angiotensin II. Finally, NO can suppress hepatic erythropoietin (EPO) expression, reducing haemoglobin levels and thus the haematocrit.

A number of recent studies have sought to identify the mechanisms underpinning the effect of dietary nitrate on hypertension. Wilcox postulated that during hypertension renal oxidative stress decreases NO bioavailability through the production of reactive oxygen species, and suggested that the kidney may mediate the effects of dietary nitrate on blood pressure 109. The kidneys regulate long‐term maintenance of blood pressure via the release of vasoconstrictors and vasodilators, such as angiotensin II and NO, respectively 110. Gao et al. demonstrated that dietary nitrate inhibited contractions triggered by angiotensin II in the renal microvasculature, via its XOR‐catalysed reduction to NO 111. However, the effects of nitrite were shown to be partially independent of the NO‐cGMP pathway, implying an alternative means by which nitrate, via NO, can bring about vasodilation 111. This secondary mechanism was revealed to be an inhibitory action of NO on nicotinamide adenosine dinucleotide phosphate (NADPH) oxidase activity, as measured by superoxide production. Increases in angiotensin II activate NADPH oxidase, which in turn elevates levels of reactive oxygen species, and can lead to hypertension 112. Concomitant treatment of arterioles with nitrite and apocynin, an NADPH oxidase inhibitor failed to attenuate contraction relative to nitrite treatment alone, suggesting that NO inhibits NADPH oxidase activity, an effect confirmed by assessing the inhibitory impact of nitrite on renal cortex NADPH oxidase activity 112. Thus, nitrate may function via both the NO‐cGMP pathway and via effects on the renal microvasculature by inhibiting NADPH oxidase to reduce blood pressure and exert beneficial effects on hypertension. Furthermore, Ashmore et al. recently observed that dietary nitrate may reduce blood pressure by inhibiting the production of erythropoietin 113. Inorganic nitrate was found to trigger a time‐ and dose‐dependent decrease in the haematocrit and circulating haemoglobin and erythropoietin concentrations. Nitrate also decreased hepatic expression of erythropoietin, which was mirrored by a fall in HIF1 target gene expression suggesting an increase in hepatic oxygen availability. A third of total erythropoietin production during hypoxia may be attributed to hepatic production 114. Ashmore et al. propose that dietary nitrate may be effective as a therapeutic in any situation in which a reduction in haematocrit would be beneficial, such as polycythemia. Further, recent studies have indicated that a high haematocrit is associated with the incidence of hypertension and a pre‐hypertensive state 115, 116. Therefore, dietary nitrate could reduce blood pressure via a decrease in the expression of hepatic erythropoietin and a subsequent reduction in haematocrit. In summary, these studies together suggest that inorganic nitrate may reduce blood pressure through three distinct mechanisms (Fig. 3).

## 6 Dietary nitrate and the heart

As previously mentioned, nitrate is likely a key factor behind the cardioprotective effects of green leafy vegetables [16, 17, 18, 117]. Hypoxia is known to alter cardiac energetics and decrease mitochondrial enzyme activities in the heart 118, 119. Ashmore et al. suggest that dietary nitrate supplementation can protect the heart in hypoxic conditions by reversing hypoxia‐induced effects on respiration rates, mitochondrial complex I levels and activity and protein carbonylation, a measure of oxidative stress 120. Of particular interest was the observation that nitrate decreases arginase expression and activity, and increases l‐arginine concentrations in the heart. It is postulated that this l‐arginine could then be redirected from the arginase‐dependent production of ornithine and urea to the production of NO. Arginase activity is associated with numerous cardiac pathologies 121 and hypoxia‐induced alterations in energetics and mitochondrial dysfunction in the heart are key features of heart failure 122, 123, 124, thus dietary nitrate may have potential as a therapeutic to protect against numerous cardiac pathologies including heart failure.

## 7 Dietary nitrate, inflammation and iNOS

Obesity has long been associated with inflammation in insulin target tissues, especially adipose tissue depots, which is suggested to contribute to the pathology of the metabolic syndrome 125, 126. NO, produced by iNOS, is an important mediator of inflammation 127, 128, and iNOS expression is increased in the skeletal muscle and adipose tissue of both genetic‐ and diet‐induced models of obesity 129. Furthermore, high fat diet‐induced obese iNOS‐null mice have improved insulin sensitivity compared to obese wild‐type mice. Recent work by Yang et al. has shown inorganic nitrite can significantly reduce the mRNA levels of iNOS in activated macrophages 130. While this study did not assess the effects of dietary nitrate on iNOS expression, the effects of nitrite were found to function through NO and require XOR, suggesting similar effects would be achieved with dietary nitrate. This paper highlights the potential for dietary nitrate to alleviate aspects of inflammation mediated by iNOS in the metabolic syndrome. However, much work is still required to elucidate the interaction between nitrate and inflammation in obesity, including a focus on the effects of dietary nitrate on the expression of iNOS in insulin‐target tissues, such as skeletal muscle and adipose tissue.

## 8 Perspectives

Having initially been considered an inactive, then toxic and carcinogenic molecule, dietary inorganic nitrate is gradually being identified as a modulator of numerous aspects of the metabolic syndrome, with therapeutic potential against obesity, diabetes and hypertension. In the wake of recent work identifying the ability of nitrate to induce the browning response in adipose tissue and alter energy balance, lower blood pressure and decrease the haematocrit, the means by which nitrate protects against the metabolic syndrome are slowly being elucidated.

However, mechanistically, the picture remains incomplete. The identification of the nitrate‐nitrite‐NO pathway appeared to clarify that the bioactive effects of dietary nitrate were brought about by increasing bioavailable NO and downstream cGMP signalling. However, the recent discoveries that nitrate can inhibit the activity of both NADPH oxidase and arginase suggests the activity of nitrate is far more complex.

Furthermore, much of the in vivo work on dietary nitrate has been conducted in rodents, and there is a clear need to confirm these studies in humans to determine the therapeutic potential of dietary nitrate within the population. Nevertheless, it is evident that the use of dietary inorganic nitrate as a tool to tackle the metabolic syndrome has great potential. Dietary adjustment, with a focus on food groups high in nitrate, such as green leafy vegetables, provides an important starting point, but perhaps it is also time to reassess the attitudes to, and strict controls on, nitrate in our diets.

*The authors have declared no conflict of interest*.
